# Photoluminescent ZnO Nanoparticles and Their Biological Applications

**DOI:** 10.3390/ma8063101

**Published:** 2015-05-29

**Authors:** Zheng-Yong Zhang, Huan-Ming Xiong

**Affiliations:** 1 Department of Chemistry, Fudan University, Shanghai 200433, China; E-Mail: zhengyongzhang10@fudan.edu.cn; 2 School of Management Science & Engineering, Nanjing University of Finance & Economics, Nanjing 210023, Jiangsu, China

**Keywords:** ZnO, luminescence, cell imaging, antibacterial, drug delivery

## Abstract

During the past decades, numerous achievements concerning luminescent zinc oxide nanoparticles (ZnO NPs) have been reported due to their improved luminescence and good biocompatibility. The photoluminescence of ZnO NPs usually contains two parts, the exciton-related ultraviolet (UV) emission and the defect-related visible emission. With respect to the visible emission, many routes have been developed to synthesize and functionalize ZnO NPs for the applications in detecting metal ions and biomolecules, biological fluorescence imaging, nonlinear multiphoton imaging, and fluorescence lifetime imaging. As the biological applications of ZnO NPs develop rapidly, the toxicity of ZnO NPs has attracted more and more attention because ZnO can produce the reactive oxygen species (ROS) and release Zn^2+^ ions. Just as a coin has two sides, both the drug delivery and the antibacterial effects of ZnO NPs become attractive at the same time. Hence, in this review, we will focus on the progress in the synthetic methods, luminescent properties, and biological applications of ZnO NPs.

## 1. Introduction

Luminescent nanomaterials with potential biological applications have received extensive attention during the past years [1,2]. Previously, semiconductor quantum dots (QDs) were the most investigated luminescent nanomaterials, owing to their advantages over the organic dyes and fluorescent proteins. In general, QDs are more stable under UV light, their emission peaks are narrow, symmetric, and size-dependent, and different sized QDs can exhibit different colors under a single excitation light [3,4,5]. Among the typical QDs, CdSe, and CdTe species possess the best luminescent properties, but Cd-related compounds are harmful to both human health and the environment [6]. Therefore, in the past decade, researchers have made efforts to find candidates with low toxicity, such as dye-doped nanoparticles (NPs) [7], carbon dots [8], metal nanoclusters [9,10], upconversion luminescent NPs [11], and ZnO QDs [12].

ZnO is one of the most excellent semiconductors, with great potential for replacing the traditional Cd-related species applied in the optical and biological fields. To date, various ZnO nanostructures have been reported, including NPs, nanorods (NRs), nanotubes, nanorings, and nanoflowers. The corresponding synthetic methods, such as the sol-gel method and chemical vapor deposition have been developed, which impact the physical properties of the obtained ZnO products. For instance, the sol-gel route can produce numerous defects on the surface of the ZnO NPs, and the NPs show strong visible emission. Since the defect-related luminescence of ZnO NPs is easily destroyed by water molecules, appropriate surface modifications are required to stabilize ZnO luminescence. The luminescent ZnO NPs (also called ZnO QDs) have good photophysical properties, and their surfaces can be modified conveniently. After careful modification, ZnO NPs are very stable in aqueous solution [13] and their quantum yield (QY) can be improved to about 30% (even to 85%) [14]; their emission color can be tuned from blue to yellow [15]. Since ZnO is considered to be a safe matter generally, it has been widely used as a food additive [16], in food packaging [17], and in sunscreen products [18]. Hence, luminescent ZnO NPs, as one of the novel types of low-cost, low-toxicity, and biocompatible nanomaterials, are expected to perform better in biological applications.

In this review, we will summarize the synthetic strategies and functionalization routes for the luminescent ZnO NPs, and their applications in the detection of various analytes, as well as in biological imaging, antibacterial activity, and drug delivery. In the final section, we will discuss the challenges concerning ZnO NPs that researchers are facing currently.

## 2. Preparation and Functionalization of Luminescent ZnO NPs

### 2.1. Luminescent Mechanisms of ZnO NPs

**Figure 1:**
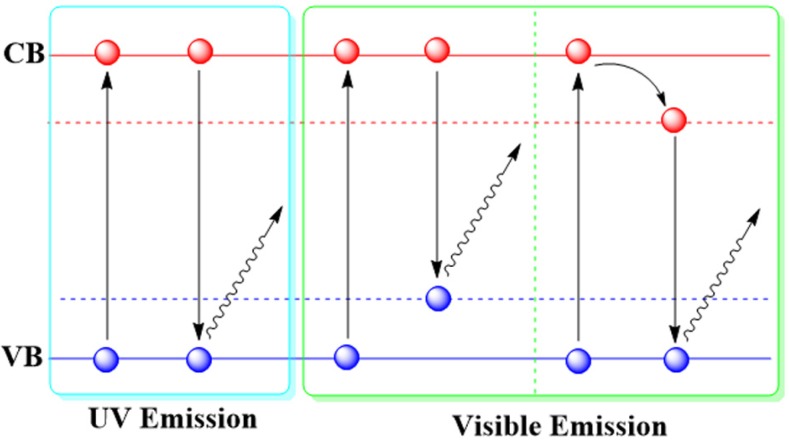
Schematic illustration of the main luminescent mechanisms for luminescent ZnO NPs. Note: valence band (VB); conductance band (CB).

Various luminescent mechanisms of ZnO NPs have been suggested, as shown in Table 1. Although these mechanisms are controversial and not clear so far, a widely accepted model is composed of two bands (Figure 1), with UV emission arising from the typical band gap transition and visible emission due to the ZnO defects. The ZnO band gap is 3.37 eV at room temperature, so that the typical UV emission is ascribed to direct exciton transition, which means the excited electron recombination with holes in the valence band (VB) or in traps near the VB produces UV emission of around 370 nm. For the visible emission, many point defects have been suggested, including oxygen vacancies, oxygen interstitials, antisite oxygen, zinc vacancies, zinc interstitials, and surface states [19]. Up to now, two popular mechanisms for this visible emission are still in debate, as discussed in our previous review [20]: one is recombination of an electron from the conduction band (CB) with a hole in a deep trap [21], and the other is recombination of holes from the VB with a deeply trapped electron [22].

**Table 1:** Recent researches about the luminescent mechanism of ZnO nanomaterials.

Materials	Size (nm)	Excitation (nm)	Emission (nm)	Luminescent mechanism	Ref.
ZnO phosphors	50–400; 500–1500	370	510	oxygen vacancies	[23]
ZnO NPs	3.4	248	510	surface states	[24]
ZnO NPs	1–3	~308	500–530	oxygen vacancies	[25]
ZnO NPs	3.5 ± 1.5	–	Visible Emission	from conduction band to a deep trap	[21]
ZnO NPs	4.3–7	325	UV emission	exciton transition	[26]
Polyether-Grafted ZnO NPs	1–4	320~365	450–570	oxygen vacancy	[27]
ZnO@PMAA–PMMA NPs	2.1	UV light	~420	ZnO and organic species	[14]
ZnO NPs and NRs	NPs: 2–6 NRs: 2–4 × 6–50	360–420 280–360	440 580	related to the ligands on surface	[28]
ionic liquid -ZnO NPs	2.5–4	UV light	425~555	transitions to trapped levels	[29]
ZnO@polymer	ZnO 2.1–2.3 ZnO@PMMA~150, ZnO@PS~450	328–366	420–464	surface vacancies	[30]
ZnO NPs	–	–	Visible emission	defect-related	[31]
ZnO NPs	2.2–7.8	~350	450–570	defect-dependent & size-dependent	[22]
ZnO NPs	10–20	340~390	400–600	defect origins	[32]
ZnO@DDA-PVP NPs	3–6.5	UV light	395–540	quantum size effect	[33]
silane@ZnO NPs	1.6–5	UV light	402–522	surface defects	[34]
ZnO NPs	3.5 ± 0.6	325	UV emission	free exciton	[35]
ZnO NPs	20	532	660	zinc vacancy	[36]
ZnO NPs	< 50	532	793	zinc vacancy	[36]
oleate-ZnO NPs	3.6–5.2	365	500–560	intrinsic defects	[37]
ZnO NPs	3–7	320	348–362	exciton emission	[38]

Note: Ultraviolet (UV); dodecylamine (DDA); poly(vinylpyrrolidone) (PVP); polymethylacrylic acid (PMMA); polystyrene (PS); Nanorods (NRs).

### 2.2. Synthesis of Luminescent ZnO NPs

The main synthetic methods of luminescent ZnO NPs can be divided into two categories based on the luminescent mechanisms mentioned above. For the highly crystallized and purified ZnO nanostructures which show strong UV emission in general [39], pyrolysis, chemical vapor deposition, and molecular beam epitaxial growth at high temperature are usually employed [40]. For example, UV emitting ZnO QDs were recently fabricated for the first time by magnetron sputtering at room temperature [35]. In contrast, ZnO visible emission intensity is closely related to the defect concentration, so that the sol-gel technique and sonochemical synthesis at room temperature are usually employed to produce ZnO NPs with highly visible emission [41].

### 2.3. Functionalization of Luminescent ZnO NPs

The functionalization of ZnO NPs has three purposes. One is to stabilize ZnO NPs, especially in aqueous solution, because stability towards water is strictly necessary for biological applications. Another is to graft specific functional groups onto ZnO surface for targeted analyses. The third is to change or enhance ZnO luminescent properties.

In order to stabilize ZnO NPs, various shells have been employed, such as silica [34], zinc sulfide [42], organic ligands [29,43], and polymer [30]. These shells are able to prevent the NPs from spontaneous growth and aggregation, and reduce the damage to the luminescent centers by aqueous solution. It should be noted that water can exchange those unstable ligands on the ZnO NPs’ surface so as to destroy the luminescent centers [44]. There are at least three factors which should be considered carefully when the ZnO NPs are applied to biological media: (1) the employed protect shell should be biocompatible to the tissues and cells; (2) the luminescence emission of ZnO NPs should not be covered by the autofluorescence of the biological background; and (3) the luminescence of ZnO NPs should not be quenched by surface modification. To overcome these difficulties, our group proposed a two-step copolymerization route for the preparation of core-shell ZnO NPs with stable luminescence in aqueous solution [45]. The polymer shell contains a hydrophobic internal layer and an external hydrophilic layer, which protects the ZnO cores and makes the NPs water dispersible, respectively.

As for the specific functionalization, silane-based polymers [46,47] and hyperbranched polymers [48] were employed frequently. On the one hand, the silica or hydrophobic ligands protect the ZnO luminescent centers. On the other, the polymer dendrons or hydrophilic shells not only render good solubility in water, but also provide terminal groups like amines, carboxylic acids, and thiols for grafting specific groups by conjugation chemical reactions. For instance, 3-[] trimethoxysilane can be used for post surface modification of silica coated ZnO NPs by introducing amino groups [49], and then conjugating with bioactive molecules for biometrics.

Doping metal ions into ZnO NPs is an effective method to modify ZnO luminescence. In our previous research, Mg^2+^ was doped into the lattice of ZnO NPs and the luminescence was adjusted from blue to yellow with highly improved efficiency [50]. Co^2+^, Ni^2+^ and Mn^2+^ ions have also been introduced into ZnO NPs to obtain magnetic semiconductors [51,52], while Tb^3+^, Er^3+^ doped ZnO nanopowders exhibit intense visible emission and near infrared (NIR) up-converted emission [53,54].

## 3. Luminescence Sensors Based on ZnO NPs

### 3.1. Detection of Metal Ions

As shown in Table 2, various researches about luminescent sensors of ZnO NPs have been presented. For example, Cu^2+^ is a well-known environmental pollutant but a necessary trace element in biological systems. Luminescent ZnO NPs have been employed as Cu^2+^ sensors based on the quenching of their luminescence. Ng *et al*. [55] found this novel, turn-off luminescent assay for Cu^2+^ ions had highly repeatable and reliable performances, and the limit of detection (LOD) was measured to be 7.68 × 10^−^^7^ M.

**Table 2:** Typical researches about luminescent sensors of ZnO NPs.

Materials	Target	LOD	Strategy	Ref.
ZnO NPs	Cu^2+^	7.68 × 10^−7^ M	PL turn-off	[55]
Imine-linked-ZnO NPs	Co^2+^	4 × 10^−10^ M	PL turn-off	[56]
APTES-ZnO NPs	dopamine	1.2 × 10^−8^ M	PL turn-off	[57]
APTES-ZnO NPs	picric acid	2.86 × 10^−6^ M	PL turn-off	[58]
Metal-organic ZnO NPs	phosphate	5.3 × 10^−8^ M	PL turn-on	[59]
silane-ZnO NPs	aldehyde	–	PL switch	[60]
Cd-doped ZnO NPs	bisphenol A	1.31 × 10^−8^ g·mL^−1^	immunosensor	[61]
ZnO NPs	carbohydrate antigen	2.5 × 10^−1^ U·mL^−1^	immunosensor	[62]

Note: limit of detection (LOD); photoluminescence (PL); (3-aminopropyl) triethoxysilane (APTES).

The corresponding quenching mechanism of the luminescent ZnO NPs for the sensing of Cu^2+^ ions was observed to be more profound, suggesting a dynamic quenching between the luminescent ZnO NPs and the Cu^2+^ ions in this work. This is based on the experimental results that higher temperature (30 °C instead of 25 °C) promotes greater kinetics and would definitely cause better interaction leading to greater quenching efficiency. Recently, Sadollahhani *et al*. [63] presented a colorimetric disposable paper based on ZnO@ZnS core shell NPs for detection of Cu^2+^ ions in aqueous solution. This test paper was based on a simple colorimetric response between Cu^2+^ ions with the core-shell NPs, so a highly selective sensor was needed to detect Cu^2+^ ions with a visual LOD as low as 1.5 × 10^−^^5^ M. The mechanism of this colorimetric sensor was suggested to be that, once the Cu^2+^ is transferred on this sensor, cation exchange begins at the interface between the ZnO@ZnS core sell NPs surface and the Cu^2+^ aqueous solution, and hence the Cu^2+^ leads to the formation of CuS and the color changing from white to brown. In another application, Sharma *et al.* [56] coated organic receptors (bearing imine linkages) on ZnO nanocrystals at room temperature, and the obtained imine-linked ZnO NPs were sensitive to Co^2+^ selectively. The reason is that the imine-linked receptor coated on the ZnO NPs surface is responsible for the selectivity in recognition of Co^2+^. The LOD of this approach was estimated to be 4 × 10^−^^10^ M.

### 3.2. Detection of Organic Molecules

Dopamine (DA), as one the of significant catecholamine neurotransmitters and derived from the amino acid tyrosine, plays a crucial role in normal homeostasis and clinical diagnosis. The abnormal content of DA may result in several diseases and neurological disorders, such as schizophrenia, Parkinson’s, and Alzheimer’s diseases [64]. A simple luminescence method for DA detection has been developed based on (3-aminopropyl) triethoxysilane (APTES) capped luminescent ZnO NPs [57]. The luminescence of ZnO NPs can be quenched by DA with high selectivity and sensitivity, and the relative luminescence intensity was linearly proportional to the concentration of DA within the range 5 × 10^−^^8^–1 × 10^−^^5^ M, with the LOD of 1.2 × 10^−^^8^ M. The sensing mechanism was suggested to be due to the luminescence quenching effect caused by the electron transfer from ZnO NPs to oxidized dopamine-quinone. 2,4,6-trinitrophenol (picric acid, PA) is one of the most toxic compounds, and it can cause anemia, headache, and liver injury. Singh *et al*. [58] synthesized water stable APTES capped ZnO NPs which can be quenched by PA with high selectivity and sensitivity (LOD = 2.86 × 10^−^^6^ M). The quenching mechanism could be attributed to the PA binding on the surface of APTES capped ZnO NPs, which traps the excited electron by electron transfer mechanism.

Unlike the above-mentioned luminescence turn-off detection mode, Zhao *et al*. [59] introduced a novel luminescence turn-on assay for phosphate. Before detection, the amine-zinc interactions and electrostatic interactions between the negatively charged metal-organic frameworks and the positively charged ZnO NPs resulted in the luminescence quenching of ZnO NPs, along with the formation of a flower-like complex. After introducing phosphate ions into the complex system, the burst of phosphate ions could inhibit the quenching effects, and recover the luminescence of ZnO NPs. The present luminescent sensing strategy exhibits good sensitivity in the range of 5 × 10^−^^7^–1.2 × 10^−^^5^ M, and the LOD was measured to be 5.3 × 10^−^^8^ M.

Jana *et al*. [60] reported another strategy to control photostability of silane modified ZnO NPs for selective detection of aldehyde groups which commonly exist in biomolecules and bio-intermediates. The aldehyde groups could form imine with the amino-functionalized ZnO NPs, leading to luminescence quenching of ZnO NPs under UV light. ZnO luminescence was reduced by 54% and 71% from the maximum intensity after 10 min of UV exposure, when the o-phthalaldehyde (OPA) concentrations are 5 × 10^−^^5^ M and 5 × 10^−^^4^ M, respectively. In contrast, only 29% reduction from the maximum luminescence intensity of ZnO NPs was observed after 10 min of UV exposure without OPA.

As a new technique, a fluorescence-linked immunoassay (FLISA) based on luminescent Cd-doped ZnO NPs was utilized for the determination of bisphenol A (BPA) [61]. The BPA antibodies were coupled to the water-soluble luminescent Cd-doped ZnO NPs, then they were employed to FLISA analysis. Furthermore, this immunosensing is based on a competitive luminescence signal change due to the 4,4-bis (4-hydroxyphenol) valeric acid (BVA), which is also an antigen like BPA. When the concentrations of BVA and ZnO NPs labeled antibodies were fixed values, respectively, the BVA was coated at a substrate, and then the different concentrations of BPA and fixed concentrations of ZnO NPs labeled antibodies were added to the substrate. BPA enabled competitive reaction in order to reduce the reaction between BVA and antibodies, and finally, different luminescence signals could be obtained and reflected the concentration of BPA. The experimental results included the linear working range of 2.08–33.03 × 10^−^^8^ g·mL^−^^1^ and the LOD of 1.31 × 10^−^^8^ g·mL^−^^1^ in this work.

### 3.3. Detection of Proteins

Conjugation of selective receptor molecules such as antibodies and aptamers to the surface of luminescent ZnO NPs has been employed for detection of proteins. Gu *et al*. [62] reported that they developed a sandwich-type sensitive immunoassay to detect carbohydrate antigen 19-9 (CA 19-9), by using ZnO NPs as both electrochemical and luminescent labels. CA 19-9 is a preferred label for pancreatic cancer, which is a highly lethiferous sarcomata and difficult to be diagnosed early in current clinical medicine. Through the immunoreaction of ZnO labeled CA 19-9 antibodies and antigens, the immunological recognition of CA 19-9 was by detection of the amplified signals of an intrinsic luminescence of the labeled ZnO or by the square wave stripping voltammetry. The luminescent method had a detection range of 1–180 U·mL^−^^1^ and a LOD of 2.5 × 10^−^^1^ U·mL^−^^1^, while the electrochemical assay exhibited a dynamic range of 0.1–180 U·mL^−^^1^ with a LOD of 4 × 10^−^^2^ U·mL^−^^1^.

## 4. Biological Imaging by ZnO NPs

### 4.1. Fluorescence Biological Imaging

Semiconductor QDs were used for the first time for biological imaging in 1998, and this opened a prelude to the biological applications of QDs [65,66]. However, the traditional QDs, like CdSe and CdTe, have encountered potential safety concerns *in vivo* [67]. Luminescent ZnO NPs, due to their special advantages, such as bright emission, low cost, and good biocompatibility, were reported to be a better candidate in biological applications [68,69], as we summarized in Table 3. For example, we prepared stable luminescent ZnO NPs and applied them to cell imaging successfully for the first time in 2008 [45]. Later, the luminescent ZnO NPs were injected into mice, and exhibited visible emission on the mice skin under UV light [70].

Despite these advances in bioimaging applications, there are still challenges in front of us. First, the luminescent ZnO NPs have a relatively broad emission band, resulting in the overlap of their fluorescence spectra with the biological autofluorescence [3]. Secondly, since the ZnO bandgap (3.37 eV at room temperature) locates in the UV region, UV light is necessary to excite ZnO luminescence, and most bioimaging applications in the literature are based on single photon UV excitation [27]. However, UV light is not suitable for deep tissue imaging *in vivo* due to the reduced penetration depth, absorption, and scattering of optical signals, and, in fact, it can only penetrate the animal skin by several millimeters. In addition, UV light is harmful to cells, tissues, and live animals [71,72]. Therefore, multi-photon or fluorescence lifetime imaging may be solutions to the above problems.

**Table 3:** Typical researches about biological imaging of ZnO NPs.

Materials	Size (nm)	Models	Imaging modality	Ref.
ZnO@polymer NPs	3–4	human hepatoma cells	disk scanning confocal microscopy	[45]
ZnO NPs	2–6	NIH/3T3 cells	disk scanning confocal microscopy	[69]
ZnO@PMAA-co-PDMAEMA NPs	4	COS-7 cells	laser scanning confocal microscopy	[73]
ZnO@silica NPs	ZnO 4–7 ZnO@SiO_2_ ~50	NIH/3T3 cells	disk scanning confocal microscopy	[49]
ZnO@polymer NPs	3–4	BALB/c mice	fluorescence microscopy	[70]
Gd-doped ZnO QDs	3–6	HeLa cells	confocal laser scanning microscopy	[74]
ZnO-Au@PEG NPs	45–98	B16F10 cells	confocal laser scanning microscopy	[68]
CdSe(S)/ZnO QDs	2–4	*S. oneidensis*	confocal microscopy	[75]
ZnO@silica NPs	2–5	Hela cells	Laser scanning confocal microscopy	[76]
ZnO nanocrystals	< 100	KB cells	nonresonant nonlinear optical microscopy, SFG SHG FWM	[77]
ZnO NPs	15–30	skin tissue/cellular architecture	multiphoton microscopy, SHG	[78]
ZnO NPs	21	skin	nonlinear optical microscopy, SHG	[79]
ZnO NPs	2–200	plants tissues cell implosion	nonlinear optical microscopy, SHG	[80]
ZnO NPs	10–300	blood cells of zebrafish; roots and shoots of *Arabidopsis* plants	nonlinear imaging, SHG	[81]
ZnO NPs	26–30	human skin and rat liver	fluorescence lifetime imaging	[82]

Note: second harmonic generation (SHG); sum frequency generation (SFG); four wave mixing (FWM).

### 4.2. Multiphoton Biological Imaging

**Figure 2:**
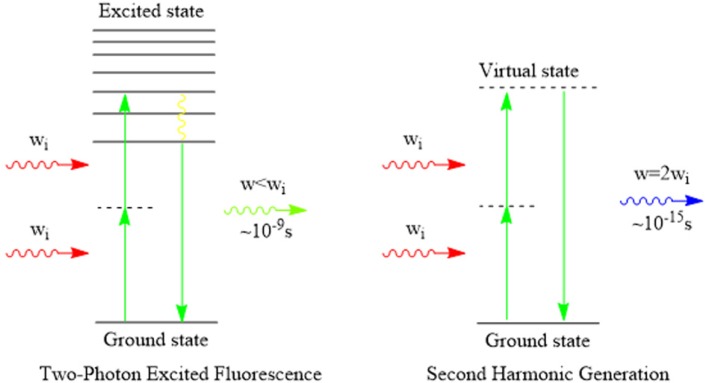
Two-photon excited fluorescence and SHG. Reprinted with permission from reference [84]. Copyright 2010, Proceedings of the National Academy of Sciences of The United States of America.

Two-photon imaging or multiphoton imaging has advantages in the deeper imaging inside tissues and the reduced photo-toxicity of NIR light. A two-photon process occurs when a molecule is excited by absorbing two photons simultaneously, as shown in Figure 2. Two-photon excitation or multiphoton fluorescence imaging using NIR excitation allows ZnO NPs to overcome the bottleneck of high energy UV excitation wavelengths. Unfortunately, the directly two-photo excited luminescence from ZnO NPs has not succeeded yet, especially in biological imaging, because an efficient two-photon excited luminescence is correlated with two-photon resonance and limited to specific wavelengths [83]. Interestingly, ZnO NPs exhibit non-linear properties which give us another choice.

Second harmonic generation (SHG) is not a fluorescence process such as usually occurs in combination with two-photon excited fluorescence. Unlike fluorescence involving fluorescence radiation from an excited state, SHG occurs when two photons interact with an asymmetric material in a scattering process to generate a single photon at twice the energy (*i.e.*, at half the excitation wavelength) [84], as shown in Figure 2. Since it does not have excited state, SHG is an ultrafast optical process with virtually no measurable lifetime. The common nonlinear materials, including the biologically significant molecule collagen, and certain membrane-bound dyes, or specially designed nonlinear molecular probes, have been reported [85]. The ratio of SHG to autofluorescence intensity in mammalian skin has been used as an indicator of skin ageing [86]. In previous reports, SHG imaging provides plenty of advantages [84,87,88]: (1) SHG is an inherent signal and does not need the external dyes; (2) the imaging signal and background are better than single photon UV excited autofluorescence imaging; (3) nonlinear excitation allows three-dimensional (3D) resolution *in vivo*; (4) due to the fact that SHG is a nonresonance nonlinear process, it does not require conventional phase matching compared with the traditional two-photon bioimaging; (5) SHG does not hurt cells and tissues owing to its nonradiative decay pathway; and (6) SHG emission wavelength can be inter changed with the excitation wavelength, allowing spectral separation between signals from the targeted molecule and other autofluorescence molecules by a proper choice of the excitation wavelength.

**Figure 3:**
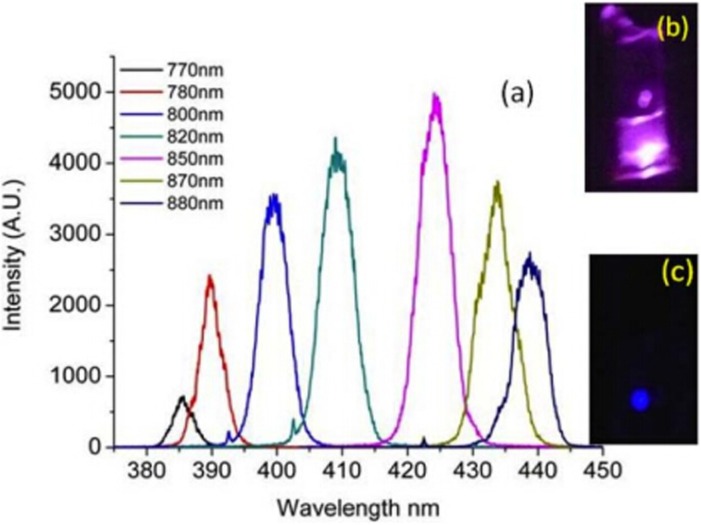
(**a**) Second harmonic spectral profile from ZnO NPs under various laser excitations. (**b**) SHG emission from ZnO NPs dispersed in water and irradiated by 840 nm laser source. (**c**) SHG emission at 420 nm with a blue filter. Reprinted with permission from reference [80]. Copyright 2012 Wiley-VCH Verlag GmbH & Co. KGaA.

ZnO NPs have a noncentrosymmetric structure, so they can be used as nonresonant nonlinear optical probes for bioimaging applications by the SHG technique. As shown in Figure 3, the SHG emission spectra from the ZnO NPs can be observed with tunable wavelengths of the incident laser [89], and the SHG efficiency is unaffected by the dispersion of ZnO NPs in water [80]. The SHG signal from ZnO NPs is spectrally well-defined and is dependent on the full-width at half maxima of the incident laser pulse. These results exhibit four advantages of ZnO NPs for SHG imaging: (1) this spectral width is significantly narrower than full-width at half maxima of molecular dyes, fluorescent proteins, or traditional QDs; (2) the intensity variation is induced by the change in the input intensities and the change in the grating efficiency; (3) the SHG signal does not blink or photobleach like most of the fluorescent QDs, and is observed to be stable over several hours of illumination [90]; and (4) it can effectively avoid the interference of autofluorescence by choosing the appropriate excitation laser source.

**Figure 4:**
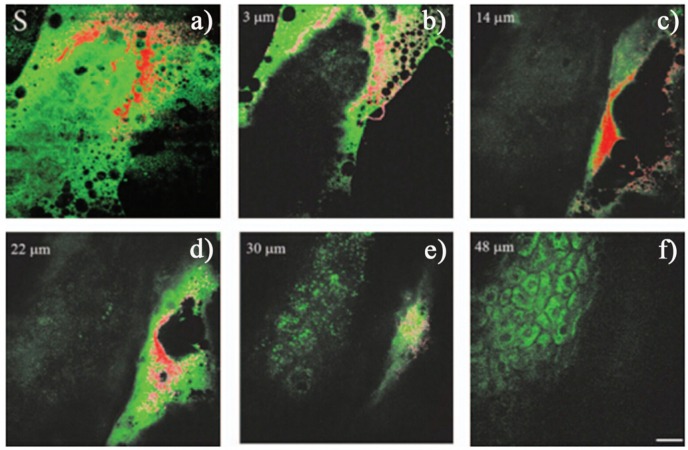
Overlaid multiphoton microscopy images of human skin *in vivo* (green) and ZnO NPs distribution (red) 4 h after its topical application. *En face* optical sections of the skin are displayed from (**a**–**f**) at depths of 0, 3, 14, 22, 30 and 48 μm from the skin surface designed S, respectively. Scale bar 20 μm. Reprinted with permission from reference [78]. Copyright 2008 Society of Photo Optical Instrumentation Engineers.

In a biological system, e.g., in skin, the autofluorescence on excitation in the range of 340~380 nm are mostly attributed to nicotinamide adenine dinucleotide/nicotinamide adenine dinucleotide phosphate (NAD [P] H), flavin adenine dinucleotide (FAD), and porphyrins, which provide about 75%, 25%, and 2%, with center wavelengths at 450 nm, 520 nm, and 625 nm, respectively [91]. Zvyagin *et al*. [78] found that the emission of ZnO NPs could be separated from the autofluorescence in skin using an appropriate spectral band. Figure 4 shows the *in vivo* images of human skin treated by the ZnO NPs. Overlaid multiphoton images of human skin *in vivo* and ZnO distribution, 4 h after its topical treatment, are colored green and red for human skin and ZnO NPs, respectively. The signals from ZnO can be clearly observed on the skin autofluorescence background, especially pronounced on the top-most layers of the skin, and the ZnO NPs’ localization in the skin folds and dermatoglyph (14~30 μm). The result confirmed the potential prospect of ZnO NPs for deep imaging inside tissues by using SHG.

### 4.3. Fluorescence Lifetime Imaging

Fluorescence lifetime imaging (FLIM) is another useful biological imaging technique, which is based on measuring the lifetime of the fluorescent molecule which is used to stain the biological sample, and it can be measured in any phase: gas, liquid, solid, or any combination of these phases [85]. As shown in Table 4 and Table 5, compared with the lifetime of the autofluorescence background from cells and tissues, ZnO QDs have an alterable luminescence lifetime after excitation, and this could be an advantage for time-gated imaging, as long as the appropriate spectral emission bands and synthesis methods are used. In general, the exciton emission has a short lifetime, while the defect related emission shows a long lifetime. In an attempt to investigate the FLIM for ZnO NPs, first of all, the appropriate experimental condition should be selected to avoid overlapping with the autofluorescence. Roberts *et al*. [86] found that the shorter fluorescence lifetime for ZnO nanomaterials considerably overlapped with that of the dominant NAD (P) H autofluorescence. However, the separation is applicable with longer lifetime measured by a pulse laser.

**Table 4:** Typical researches about the autofluorescence lifetime of the biological system.

Fluorophore	Excitation (nm)	Emission (nm)	Lifetime (ns)	Ref.
phenylalanine	240–270	280	7.5	[85,92]
tyrosine	250–290	300	2.5	[85,93]
tryptophan	250–310	350	3.03	[85,94]
flavin mononucleotide	420–500	520–570	4.27–4.67	[85,95]
riboflavin	420–500	520–570	4.12	[85,96]
protoporphyrin IX	400–450	635, 710	up to 15	[85,97]
lipofuscin	340–395	540, 430–460	1.34	[85,98]
NAD (P) H free	300–380	450–500	0.3	[85,97]
NAD (P) H free	730–800 (2P)	450–460	0.3–0.7	[86,99]
NAD (P) H bound	300–380	450–500	2.0–2.3	[85,97]
NAD (P) H bound	730–800 (2P)	450–460	2.5–3.0	[86,99]
FAD	420–500	520–570	2.91	[85,97]
FAD	~800 (2P)	525–550	2.3–2.8	[86,100]
FAD bound	420–500	weak in 520–570	<0.01	[85,98]
FAD bound	~800 (2P)	525–550	0.04–0.4	[86,100]
Melanin	300–800	440, 520, 575	0.1/1.9/8	[85,97]
Melanin	730–830 (2P)	~550	0.1–0.2, 0.7–1.4	[86,101]
Elastin	290–340	420–460	0.2–2.5	[82,97]
Elastin	760–830 (2P)	475–575	0.26, 1.96	[86,102]
Keratin	750–800 (2P)	382, 450–500	1.4	[86,103]
Collagen	280–350	370–440	≤5.3	[85,97]
Collagen, SHG	730–880 (2P)	1/2 of excitation wavelength	No lifetime	[86,87]

Note: two-photon excitation (2P); second harmonic generation (SHG).

**Table 5:** Typical researches about the luminescence lifetime of ZnO NPs.

Materials	Size (nm)	Excitation (nm)	Emission (nm)	Lifetime (ns)	Ref.
ZnO NPs	~4	248	510	~1000	[24]
ZnO NPs	1	308	visible (500–530)	920	[25]
ZnO NPs	3	308	visible (500–530)	1340	[25]
ZnO NPs	~4	380	460	2, 9	[28]
ZnO NPs	–4	320	600	9, 60, 1850	[28]
ZnO NPs	26–30	405	–	21, 478, 2500	[82]
ZnO NPs	3–4	320–340	530	3066, 1000	[104]
ZnO NPs	3~4	320–340	510	2750, 885	[104]
ZnO NPs	3–4	320–340	490	729, 109	[104]
ZnO QDs	3–7	320	348–362	(9.8~29.6) × 10^−3^	[38]
ZnO NPs	–	350	~370	~0.14, 3.12	[105]
ZnO NPs	–	730 (2P)	~430	~0.13, 1.56	[105]

Note: two-photon excitation (2P).

**Figure 5:**
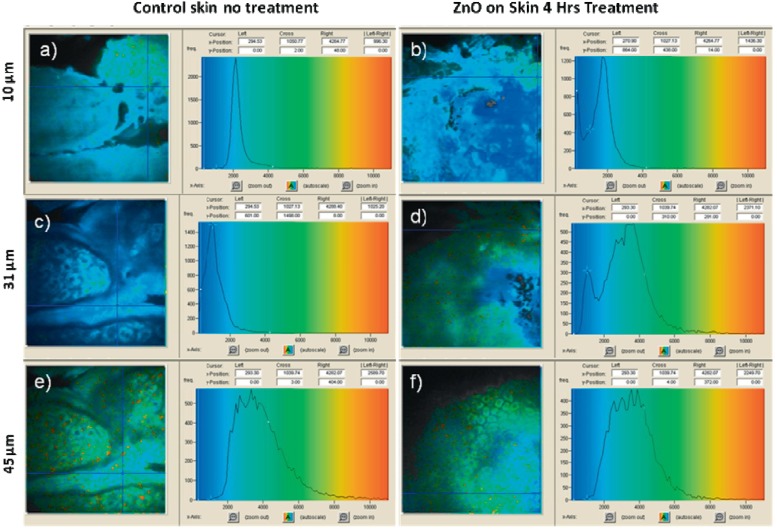
*In vivo* control and treated skin after exposure of ZnO for 4 h intensity versus life times for ZnO, stratum corneum, and viable epidermis. 740 nm excitation, channel 1 (350~415 nm), field of view 175:175 μm. (**a**) Stratum corneum control, (**b**) stratum corneum after ZnO treated for 4 h, (**c**) first layer of viable epidermis control, (**d**) first layer of viable epidermis after ZnO treated for 4 h, (**e**) viable epidermis control and (**f**) viable epidermis after ZnO treated for 4 h. Reprinted with permission from reference [82]. Copyright 2008 Wiley-VCH Verlag GmbH & Co. KGaA.

ZnO NPs have been employed to fluorescence lifetime imaging in human skin [82], as shown in Figure 5. The skin was treated with ZnO NPs for 4 h, then the skin was visualized with multiphoton imaging at different depths below the skin surface, as was the untreated skin. At 10 μm from the surface of human skin, an additional peak at 270 ps can be observed in the treated sample but cannot be seen in the control (Figure 5a,b). This peak represents the short lifetime of ZnO NPs as the dark blue region in the false colored image. At 31 μm depth, there is a peak at about 3000 ps apparent in the treated skin but not in the control (Figure 5c,d). However, no signals from ZnO are found in the treated skin at 45 μm depth. This research confirmed ZnO FLIM for human skin can reach several tens of micrometers in depth.

## 5. Antibacterial Activity of ZnO NPs

**Figure 6:**
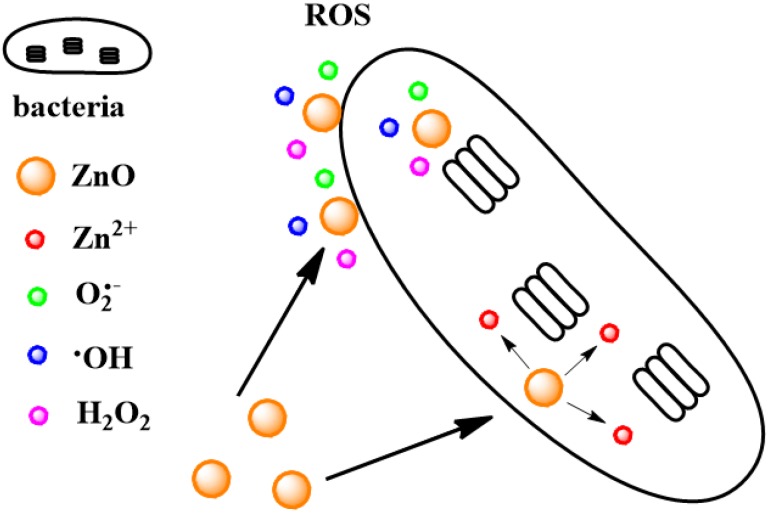
Schematic illustration of antibacterial activity of ZnO NPs.

ZnO is currently listed as a generally recognized safe material by the US Food and Drug Administration (21CFR182.8991) [106]. Yang *et al*. [107] recently investigated the toxicity and biodistribution of aqueous ZnO QDs in mice, and the results showed that no haemolysis occurred even at a high concentration of 1600 μg·mL^−1^ *in vitro* haemolytic assay, which demonstrated that the QDs-PEG (polyethylene glycol, PEG) displayed good blood compatibility. However, ZnO NPs exhibit antibacterial properties against a range of both gram-positive and gram-negative bacteria through a cytotoxic mechanism, and have attracted heightened research interest, as shown in Table 6. It has been suggested that the mechanism by which ZnO NPs demonstrate antibacterial activity may involve the accumulation of NPs in the outer membrane or cytoplasm of bacterial cells [108], and may be attributed to the excess reactive oxygen species (ROS) [109] and the release of Zn^2+^ [110,111], as shown in Figure 6. ROS are generated from activated ZnO NPs and usually in the light, which include superoxide (O_2_–), hydroxyl radical (–OH), and hydrogen peroxide (H_2_O_2_). Compared with O_2_– and –OH, H_2_O_2_ is often considered the main harmful factor, because it can penetrate the bacteria easily due to relatively weaker electrostatic interaction between ions and bacteria [112]. For the cytotoxic of Zn^2+^, Applerot *et al*. [113] reported that no significant effects on the viable count of *E. coli* or *S. aureus* were observed even when 25 mg·L^−1^ of Zn^2+^ was employed in the experiment. In contrast, Bellanger *et al*. [110] reported that ZnO QDs and ZnCl_2_ were more toxic towards *E. coli* MG1655 and *C. metallidurans* CH34, and the IC_50_ for *E. coli* MG1655 were 1.6 × 10^−5^ M and 2.7 × 10^−5^ M for ZnCl_2_ and ZnO QDs, respectively, while the IC_50_ for *C. metallidurans* CH34 were 2.8 × 10^−4^ M and 1.2 × 10^−3^ M for ZnCl_2_ and ZnO QDs, respectively. And thus the toxicity is mainly from Zn^2+^. We think there are two factors, at least, related to the conflicting conclusion of Zn^2+^ ion toxicity: one is that the different bacteria has different intrinsic, the other is that Zn^2+^ is a nutrient for the bacteria when it is in a lower concentration, but is antibacterial at high concentrations. So the suitable Zn^2+^ concentrations are different when different biological systems are studied.

**Table 6:** Typical researches about antibacterial activity of ZnO NPs.

Materials	Size (nm)	Models	Treatment	Antibacterial result	Antibacterial mechanism	Ref.
ZnO	~480	*B. subtilis CB310*	10 ppm	90% growth reduction of *B. subtilis*	ROS	[114]
ZnO	~480	*E. coli DH5α*	1000 ppm	48% growth reduction of *E. coli*	ROS	[114]
Acetate-ZnO QDs	3~5	*E. coli*	2.5 mM in light	MIC	ROS	[115]
Acetate-ZnO QDs	3~5	*E. coli*	3 mM in dark	MIC	ROS	[115]
Nitrate-ZnO QDs	4–7	*E. coli*	6 mM in light, 30 mM in dark	MIC	ROS	[115]
Nitrate-ZnO QDs	4–7	*E. coli*	30 mM in dark	no significant bacterial growth inhibition	ROS	[115]
ZnO NPs	6.8 ± 2	*E. coli* and *S. aureus*	0.1 mg·mL^−1^ for 3 h	inactivation of *E. coli* by 99.8%, that of *S. aureus* by 98%	ROS	[113]
ZnO NPs	260 ± 40	*E. coli* and *S. aureus*	0.1 mg·mL^−1^ for 3 h	inactivation of *E. coli* by 99.5%, that of *S. aureus* by 89%	ROS	[113]
ZnO NPs	800 ± 300	*E. coli* and *S. aureus*	0.1 mg·mL^−1^ for 3 h	inactivation of *E. coli* by 87%, that of *S. aureus* by 68%	ROS	[113]
ZnO NPs	800 ± 300	*E. coli* and *S. aureus*	5 mg·mL^−1^ for *E. coli* and 10 mg·mL^−1^ for *S. aureus.*	MIC	ROS	[113]
ZnO NPs	~20	*E. coli* 11634	543 nm, 1000 lux light for 24 h	<20 bacteria count (CFU)	hydrogen peroxide (H_2_O_2_) ROS	[116]
Ag-ZnO composite	~64	*S. aureus* and* GFP E. coli*	550 μg·mL^−1^ for *E. coli* and 60 μg·mL^−1^ for *S. aureus*	MIC	ROS and the release of Ag^+^, Zn^2+^	[117]
ZnO QDs	~4	*E. coli* MG1655 and *C. metallidurans* CH34	1.6 × 10^−5^ M for *E. coli* MG1655 and 2.8 × 10^−4^ M for *C. metallidurans* CH34	IC50	mainly result from the liberation of Zn^2+^ cations from the ZnO QDs	[110]
crystal violet-ZnO silicone polymers	ZnO NPs 3.3 ± 1.1	*S. aureus*	1 h incubation in dark or 1 h in standard hospital light	Significantly reduced under dark or 3.36 log kill in light	PDT	[118]
					ROS	
crystal violet-ZnO silicone polymers	ZnO NPs 3.3 ± 1.1	*E. coli*	6 h in the dark or in standard hospital light	1.41 log kill in dark or below the detection limit	PDT	[118]
					ROS	
CdO-ZnO composite	~27	*E. coli*	10 μg·mL^−1^	MIC	ROS and Zn^2+^, Cd^2+^ release	[119]
CdO-ZnO composite	~27	*S. aureus*	10 μg·mL^−1^	MIC	ROS and Zn^2+^, Cd^2+^ release	[119]
CdO-ZnO composite	~27	*P. aeruginosa*	15 μg·mL^−1^	MIC	ROS and Zn^2+^, Cd^2+^ release	[119]
CdO-ZnO composite	~27	*P. vulgaris*	20 μg·mL^−1^	MIC	ROS and Zn^2+^, Cd^2+^ release	[119]
CdO-ZnO composite	~27	*K. pneumonia*	20 μg·mL^−1^	MIC	ROS and Zn^2+^, Cd^2+^ release	[119]
CdO-ZnO composite	~27	*Bacillus spp.*	15 μg·mL^−1^	MIC	ROS and Zn^2+^, Cd^2+^ release	[119]

Note: Minimum inhibitory concentration (MIC); reactive oxygen species (ROS); colony-forming units (CFU); half maximal Inhibitory Concentration (IC_50_); photodynamic therapy (PDT); green fluorescent protein (GFP); Escherichia coli (*E. coli*); Cupriavidus metallidurans (*C. metallidurans*); Bacillus subtilis (*B. subtilis*); Staphylococcus aureus (*S. aureus*); Pseudomonas aeruginosa (*P. aeruginosa*); Proteus vulgaris (*P. vulgaris*); Klebsiella pneumonia (*K. pneumonia*).

The antimicrobial activity of the ZnO NPs was reported to have a size dependency, *i.e*., decreasing ZnO NP sizes resulted in the greater antimicrobial activity [113]. The antibacterial activity of ZnO was attributed to reactions between ZnO surface and water, and the aqueous ZnO NPs suspensions were able to produce high levels of ROS. Compared with the larger ZnO NPs, the smaller ones have both higher surface areas and larger numbers to cover target bacteria. The acetate modified ZnO QDs (3~5 nm) and nitrate modified ZnO QDs (4~7 nm) displayed great antibacterial activity against *E. coli* [115], and the differences between these two kinds of ZnO QDs suggested that the surface groups on ZnO QDs played important roles. Therefore, appropriate surface modifications are helpful to enhance the ZnO antibacterial activity, such as crystal violet-coated ZnO encapsulated silicone polymers [118], Ag-ZnO nanocomposite [117], and CdO-ZnO nanocomposite [119]. Adams *et al*. [114] reported that when *E. coli* DH5α and *B.* *subtilis* CB310 were treated with the similar ZnO nanomaterials, respectively, different antibacterial activities were found. This result revealed that the tolerance and surface charge of bacteria are also important factors for the ZnO antibacterial activity [120].

## 6. Drug Delivery by ZnO NPs

**Figure 7:**
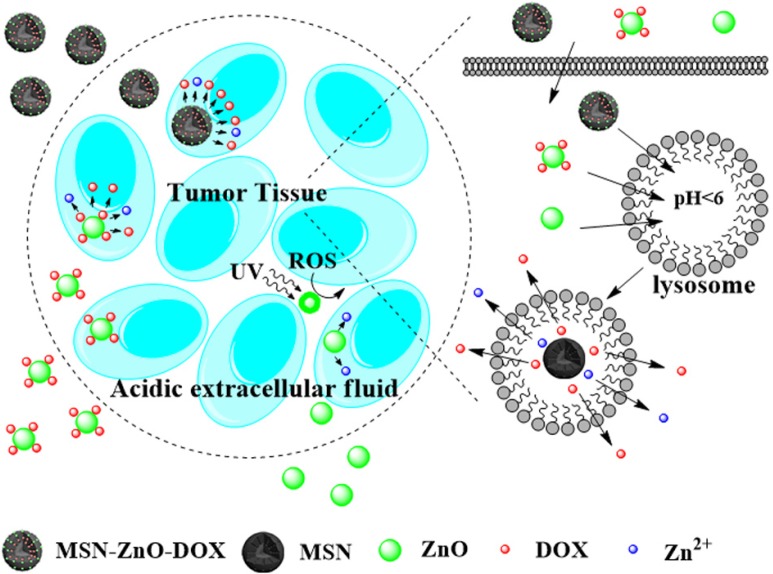
Schematic illustration of the DOX delivery from the ZnO NPs.

Cancer is a serious threat to human health, and it is the second leading cause of death in the USA accounting for about 25% of all deaths [121]. However, current anticancer chemotherapies often show toxic adverse effects and low efficacy due to the failure to differentiate between cancerous and normal cells by the drug itself, as well as the development of drug resistance. Drug delivery systems (DDSs) based on nanotechnology exhibit great potential in anticancer treatment and have been employed to deliver anticancer drugs to the target tissues. Considering the extracellular mildly acidic environment in the solid tumor tissues and the intracellular compartments such as endosomes and lysosomes [122,123], NPs were designed to enter cells through the cellular endocytic pathway and fuse with lysosomes [124], so that a pH-responsive DDS will be an ideal choice for cancer therapy. Fortunately, ZnO NPs can exhibit great stability in physiological condition (pH 7.4), but rapidly dissolve at pH 5~6. As shown in Table 7, a number of DDSs based on ZnO nanomaterials have been investigated and the results display their good pH-responsive profiles. ZnO NPs were used as cappers to cover the pores of mesoporous silica NPs (MSNs), and when these DDS met with acids, the ZnO NPs decomposed to release doxorubicin (DOX) molecules from MSNs [125]. However, this type of DDS has difficulty in degradation so that it cannot completely release the drugs [126,127]. Another strategy is based on loading drugs onto the simple ZnO NPs directly, and thus, when the composites meet acid the ZnO NPs decompose completely to release all the drug molecules [128]. The corresponding drug delivery mechanisms are in Figure 7. Although the stabilized ZnO NPs revealed good biocompability and low toxicity, after ZnO decomposition, Zn^2+^ ions are cytotoxic [129], so they were used directly for cancer treatment [130] or enhancing the DDS toxicity. Additionally, the ZnO NPs can produce destructive ROS, as mentioned previously in their antibacterial activity. They will be able to destroy the cancer cells directly as well, or enhance the cytotoxic effect of DDS under appropriate light. Kishwar *et al*. [131,132] recently investigated the phototoxic effect of ZnO NRs on human cells. They demonstrated that the ZnO NRs coated with drugs could exhibit the enhanced cytotoxic effect under UV irradiation as a result of the production of ROS. The photodynamic therapy (PDT) will be helpful for the tumor treatment because it can be restricted to trigger at the tumor location.

**Table 7:** Typical researches about drug delivery systems based on the ZnO NPs.

Materials	The role of ZnO	Drugs	Models	Control method	Ref.
ZnO	drugs	itself	cancerous T cells, activated human T cells	–	[130]
chitosan-ZnO NPs	carriers	DOX	–	pH	[133]
ZnO@PMAA-co-PDMAEMA NPs	carriers	DNA	COS-7 cells	–	[73]
mesoporous ZnO	carriers	DOX	–	pH	[134]
ZnO NRs	carriers	PPDME	T47D cells	PDT	[131]
ZnO-Mesoporous Silica NPs	lids	DOX	HeLa cells	pH	[135]
ZnO NPs	carriers	DOX	HeLa cells	pH	[136]
ZnO tetrapod	drugs	itself	CHO-K1 cells, HeLa cells, Vero cells and VK2/E6 cells	–	[137]
ZnO/PEG NPs	carriers	DOX	Gram-positive microorganisms	Photo-dynamic therapy	[138]
ZnO@polymer NPs	carriers	DOX	U251 cells	pH	[128]
ZnO-Au NPs	carriers	camptothecin	Hela cells	pH	[139]
ZnO@PNIPAM NPs	carriers	DOX	–	Thermal and pH	[140]
ZnO/carboxymethyl cellulose	carriers	curcumin	L929 and MA104 cell	pH	[141]
mesoporous ZnO NPs	carriers	DNA, DOX	normal lymphocyte and K562 cells	–	[142]
ZnO NRs	carriers	ALA	FM55P cells and AG01518 cells	PDT	[132]
ZnO QDs	drugs	itself	HepG2 cells	–	[143]
Curcumin/O-CMCS/n-ZnO nanocomposites	carriers	curcumin	MA 104 cells	pH	[144]
UCNP@mSiO_2_-ZnO	gatekeepers	DOX	HeLa cells and Balb/c mouse	pH	[125]

Note: poly (2-(dimethylamino) ethyl methacrylate) (PMAA-co-PDMAEMA); poly (N-isopropylacrylamide) (PNIPAM); polyethylene glycol (PEG); O-carboxymethyl chitosan (O-CMCS); upconverting nanoparticles (UCNPs); doxorubicin hydrochloride (DOX); Protoporphyrin dimethyl ester (PPDME); deoxyribonucleic acid (DNA). 5-aminolevulinic acid (ALA).

Despite DOX molecule being used as the drug in most DDS, other medicines such as toxin protein [145], short interfering RNA (SiRNA) [146], and deoxyribonucleic acid (DNA) [142], also have great potential in treatment of diseases. Zhang *et al*. [73] reported ZnO QDs as carriers for plasmid DNA delivery, such polycation-modified ZnO QDs-DNA nanocomplex which could mediate an efficient transfer of plasmid DNA into COS-7 cells. After the ZnO nanomaterials are dissolved in the acid condition in cells, the released Zn^2+^ may induce a series of harmful cellular outcomes, such as lysosomal damage, mitochondrial perturbation, ROS production, excitation of pro-inflammatory cytokine, and chemokine production [111]. Therefore, how to choose the drug and how to optimize ZnO carriers for selective destruction of tumor cells and tissues has become an urgent demand. PDT can induce nonspecific tissue and cell damage because it can be selectively illuminated at the tumor tissue. However, ZnO-related PDT DDSs were mostly based on UV irradiation, and UV light is not an ideal choice for using deep PDT in cancer treatment. Furthermore, most of the present researches are limited in the cellular level, and the evaluation of ZnO-drug nanocaomposites in live animals is still scarce.

## 7. Conclusions

In this review, the luminescent properties and biological applications of luminescent ZnO NPs have been summarized and discussed, including the luminescent mechanisms, synthesis methods, functionalized strategies, luminescent sensors, biological imaging, antibacterial activity, and drug delivery. ZnO NPs, as a kind of low-cost, low-toxic, and versatile material, have shown to have a promising future in biological applications. However, there are still challenges in front of researchers. First, the luminescent mechanisms of ZnO NPs need more deep interpretation, because many synthesis and modification routes are apt to quench ZnO fluorescence and only a few methods are able to improve or change ZnO fluorescence effectively. Secondly, ZnO bioimaging on the multiple target cells and live animals has not succeeded yet. Nonlinear fluorescence imaging and fluorescence lifetime by using ZnO NPs are investigated scarcely, and the influence factors are not fully clear. Thirdly, both antibacterial and anticancer performances of ZnO NPs have shown a bright future, but they all require plenty of work before any practical applications.
